# A Review of Advanced Localization Techniques for Crowdsensing Wireless Sensor Networks

**DOI:** 10.3390/s19050988

**Published:** 2019-02-26

**Authors:** Angelo Coluccia, Alessio Fascista

**Affiliations:** Department of Innovation Engineering, University of Salento, 73100 Lecce, Italy; alessio.fascista@unisalento.it

**Keywords:** localization, wireless sensor networks, crowdsensing, mobility, cooperation, signal processing

## Abstract

The wide availability of sensing modules and computing capabilities in modern mobile devices (smartphones, smart watches, in-vehicle sensors, etc.) is driving the shift from mote-class wireless sensor networks (WSNs) to the new era of crowdsensing WSNs. In this emerging paradigm sensors are no longer static and homogeneous, but are rather worn/carried by people or cars. This results in a new type of wide-area WSN—crowd-based and overlaid on top of heterogeneous communication technologies—that paves the way for very innovative applications. To this aim, the positioning of mobile devices operating in the network becomes crucial. Indeed, the pervasive, almost ubiquitous availability of smart devices brings unprecedented opportunities but also poses new research challenges in their precise location under mobility and dense-multipath environments typical of urban and indoor scenarios. In this paper, we review recent advances in the field of wireless positioning with focus on cooperation, mobility, and advanced array processing, which are key enablers for the design of novel localization solutions for crowdsensing WSNs.

## 1. Introduction

We are in the middle of the digital era, where more and more amazing features become available even in entry-level consumer devices (smartphones, tablets, wearable devices such as smart watches, etc.), which was inconceivable until only a decade ago. As a matter of fact, electronic (smart) devices are today ubiquitous, and the presence of different kinds of “sensors” both onboard (camera, microphone, accelerometer, radios, global navigation satellite systems (GNSS) receivers, …) and as external add-ons (e.g., via Bluetooth connectivity) is opening the doors to unprecedented applications. At the same time, since its introduction fifteen years ago in the context of radio-frequency identification (RFID) [1], the internet of things (IoT) idea has been expanding and now envisions “anytime, anywhere” connectivity with “anyone/anything”, further extended to “any path/network” and “any service” in the so-called 6A-vision [2,3].

A network of devices/subsystems with sensing, processing, and communication capabilities, which can collaboratively perform a task, is the reference scenario not only for IoT, but also intelligent transportation systems, multi-agent systems, generally smart systems (smart grids, smart cities, …). In many of such contexts, opportunistic approaches have also started to emerge, which can take advantage of existing technologies and services for additional tasks. A simple example is the use of “over-the-air” Wi-Fi and cellular signals (which were designed for communications purposes) for positioning based on the received signal power or other features (as done e.g., by Google Maps). The term mobile crowdsensing [4,5,6,7,8] has been used to identify approaches that leverage smartphone “apps” to gather different kinds of data, so creating high-value applications without requiring additional technology, hardware, or infrastructure, only proper algorithms [9,10,11]. The applications can be very diverse, from personal sensing (health indicators, position, etc.) to social sensing, where aggregate information with group dynamics is of interest, and public sensing for environmental data (noise, air pollution, etc.) or fine-grained information (parking slots, traffic jams, security/safety alarms, etc.) beneficial to the general public.

In the emerging crowdsensing paradigm sensors are no longer static and homogeneous, but are rather worn/carried by people or cars. This results in a new type of wide-area wireless sensor network (WSN)—crowd-based and overlaid on top of heterogeneous communication technologies—whose ad-hoc topology closely follows human-life dynamics. Such a novel interconnected and heterogeneous framework can serve as a critical enabler for innovative applications, in which a large number of devices cooperate and exchange locally-sensed data to solve global problems in a distributed way, so bringing the IoT idea to a new dimension. Examples are the monitoring of environmental parameters [12] such as temperature, pollution level, ambient noise level, electromagnetic exposure [13], as well as other types of variables that have a spatial profile (Obtaining capillary information on environmental variables is of utmost importance to fight pollution, global warming, generally to cope with climate changes and prevent natural disasters) [14,15,16]—a problem that is sometimes referred to as field reconstruction [17,18,19].

From the discussion above, it is evident that the positioning of devices operating in such networks becomes crucial. In principle, the enhanced capabilities of modern smart devices could give the wrong impression that the localization task can be easily accomplished simply due to the availability of a GNSS receiver onboard. However, the latter is power-hungry hence it is often kept switched-off for most of the time [20]. Furthermore, even when it is active, GNSS positioning suffers from several major limitations (e.g., degraded satellite visibility, multipath propagation, …) that prevent its application in many urban areas and indoor environments [21]. Unfortunately, such contexts are indeed among the most relevant for emerging crowdsensing applications (e.g., environmental monitoring, traffic management, …).

To overcome these limitations, a number of terrestrial technologies have been extensively researched in the past two decades. Many papers have addressed the localization problems in different scenarios, including WSNs and other network scenarios [22,23] also in mobility conditions [24]. Classical range-based or range-free techniques have been revisited in this context [25]. In [26] it is shown that range-based localization using received signal strength (RSS) data can be improved by adopting more sophisticated estimation tools; a palette of algorithms with diversified accuracy-vs-complexity trade-off can be obtained, applicable to different types of wireless signals. Indeed, as explained above, the latter are nowadays found in almost any environment of daily life, hence the possibility to opportunistically exploit them for other purposes, namely localization, is attracting more and more interest [27]. This emerging paradigm is somewhat complementary to dedicated localization technologies implementable in WSNs. Prominent examples of such technologies include RFID, ultra-wide band (UWB), and the latest long range (LoRa) positioning system. The RFID technology exploits RF electromagnetic propagation to store and retrieve data from an integrated circuit [28,29,30]. More precisely, a position estimate can be obtained by leveraging short-range communications between an RFID reader (transmitter) and a (usually passive) RFID tag [31]. RFID positioning systems have been mainly employed to identify objects in indoor environments such as supermarkets, libraries, and warehouses, thanks to their reduced cost and complexity [32], but can provide operational ranges only in the order of few meters. For much higher resolution and range capabilities, the UWB technology has been introduced [33,34]. It is based on the IEEE 802.15.4 standard and exploits a very large bandwidth (i.e., fine time resolution pulses) to perform accurate range estimation via a dedicated two-way time of arrival (TOA)-based protocol. The main advantage of such a technology resides in its ability to resolve all the different paths generated by multipath propagation. More precisely, the availability of a large bandwidth allows receivers to correctly estimate the delays of each separate path, which can be then coherently processed to improve the localization performance [35]. On the other hand, it requires WSN nodes to be equipped with enhanced computational and communication capabilities, hence it generally demands for much higher costs and power consumption. With the advent of the IoT era, another interesting localization technology called LoRa has been developed [36,37]. This system uses RSS measurements to locate targets in outdoor environments (mainly smart cities). In particular, by operating in the sub-GHz frequency band, LoRa gains improved penetration capabilities, which makes it more robust against noise and multipath when communicating over larger distances [38]. Moreover, LoRa is designed to work with low transmitting power and very limited bandwidth, which is a plus for localization in the IoT context [39,40].

As mentioned, however, the advent of ubiquitous connectivity enables a different paradigm, in which dedicated technologies are complemented (or replaced) by the opportunistic exploitation of communication signals for localization purposes. Pioneering approaches have attempted to exploit earlier cellular network technologies [41] and Wi-Fi [42], before location awareness experienced impressive momentum in the industry [43]—for instance, currently Google Maps already tries to use a mix of different signals in addition or absence of GPS, to enable everywhere-localization and improve the location accuracy. A more heterogeneous scenario is theoretically addressed in [44], where signals of opportunity are exploited in conjunction with device-to-device (D2D) communications, solving at the same time the synchronization problem, albeit in a static scenario. References [45,46] studied cases in which the transmitted power and/or the attenuation factor need to be estimated before positioning can be performed, but consider an homogeneous scenario, an assumption no longer valid in crowdsensing. In [47] a cognitive approach is proposed in which an hypothesis test decides whether the environment is homogeneous or not, and provides estimates of the unknown parameters in a fully-adaptive way; based upon the sensed scenario, a proper maximum likelihood (ML) localizer is implemented. The latter work, however, still assumes a WSN-like setting. Novel advanced methodologies are needed to tame the complexity of schemes that have to be simultaneously opportunistic, cognitive, and adaptive: for instance, inaccurate channel estimation can produce instability in some location estimators [48], and more generally the interaction between multiple inference tasks is an important issue.

The complexity of the localization task stems from the fact that the variety of emerging applications require differentiated precision, different smart devices hosting these applications carry diverse sets of sensors, and the behavioral characteristics of such sensors also depend on the operational environment. Thus, while the topic is not novel per se, the peculiarities of crowdsensing require to go beyond classical assumptions [49].

**Figure 1:**
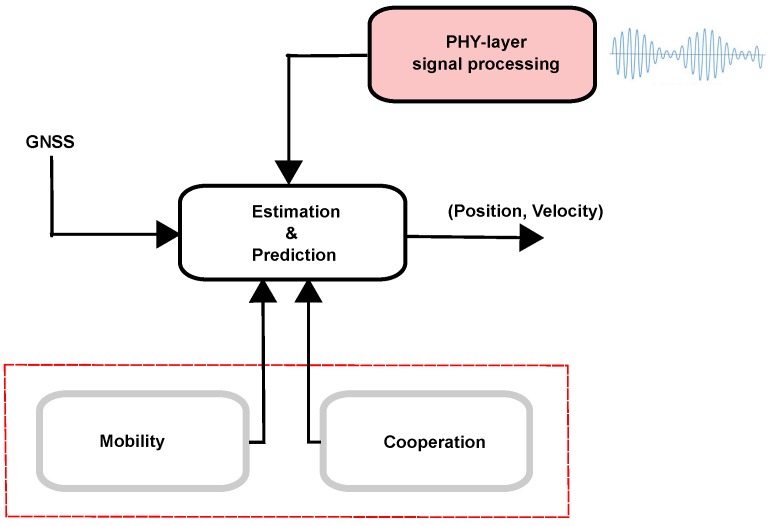
Three major ingredients for localization in emerging crowdsensing wireless sensor networks (WSNs).

In particular, we identify three major aspects on which mote-class and crowdsensing WSNs significantly differ, as shown in Figure 1. More precisely:

(i) **Cooperation**. In terms of processing power and energy constraints, mote-class WSNs are much more limited; the novel smart devices involved in crowdsensing WSNs are conversely very well-equipped in hardware, which enables sophisticated computations as well as orders of magnitude higher communications compared to the extremely low data rate of mote-class WSNs (especially when nodes are low-cost and low-power). This, in turn, paves the way for cooperative schemes that can significantly improve the accuracy and availability of localization.
(ii) **Mobility**. In mote-class WSNs, sensors are static or only slowly moving, while in crowdsensing WSNs the involved nodes are human-centric, i.e., revolve around people’s activities; as such, they are tightly connected to human life and mobility [8], including private/public transportation (car, bycicle, subway, tram, bus, …) and, through vehicle-to-anything (V2X) technologies, they can also communicate with “smart vehicles”, further increasing the potential of crowdsensing in Intelligent Transportation System (ITS) and Smart City contexts [50,51]. On the one hand, this makes the localization problem more difficult; on the other hand, the presence of inertial sensors onboard (e.g., accelerometer) makes it possible also to exploit mobility to improve the localization accuracy, for instance to cope with dense-multipath environments typical of urban and indoor scenarios;
(iii) **Array processing**. The widespread of multiple-input multiple-output (MIMO) technologies in 4G/LTE cellular networks is opening novel possibilities for localization, and will produce a revolution in future 5G mmWave scenarios in which array size significantly shrinks hence can be integrated in mobile terminals (smartphones) [52]. Interestingly, the multipath environment for mmWave is sparse in angle [53], which reduces the complexity of the localization task in this respect.

In summary, although the research on localization in WSNs has come a long way, with many contributions from both academia and industry, most literature has inherited the oversimplified and “well-behaved” (homogeneous, neutral, under control) paradigm of mote-class WSNs, while in crowdsensing WSNs the “nodes” are not part of a whole system designed for a specific purpose: as explained above, they are rather a collection of heterogeneous devices carried/worn by people in their daily life for other reasons (communication, navigation, work, gaming, …) [54], possibly interconnected with “things” as different as smart objects, external wireless add-ons, or (possibly low-cost) “gadgets” (e.g., dongles or stations with radio interfaces). In this novel kind of network scenario, it is necessary to extend localization approaches used in mote-class WSNs with more advanced techniques that have been developed in different fields, hence are currently scattered across the literature. The aim of this paper is exactly to review recent advances in the field of wireless positioning with focus on cooperation, mobility, and array processing, which as explained are key enablers for the design of novel localization solutions for crowdsensing WSNs.

The rest of the paper is organized as follows. In Section 2, we provide a review of the classical localization approaches adopted in mote-class WSNs. Then, in Section 3, we shift from the traditional assumption of static sensors to novel positioning techniques that exploit the presence of moving sensors. In Section 4, we describe the emerging topic of cooperative positioning, which allows nodes to improve their location by exchanging position-related data over the network. Then, in Section 5, we present a collection of the latest research results based on the adoption of advanced signal processing techniques. We conclude the paper in Section 6.

## 2. Review of Traditional WSN Localization Techniques

Localization in WSNs has been an active topic for more than two decades. The early progresses in the field of wireless communication technologies led to the design of small, cheap, and limited-power sensors able to interact among themselves across short distances. A large deployment of such smart and interconnected sensors provided new opportunities for monitoring different environments such as homes, industries, and cities at large [55].

Localization algorithms adopted in traditional WSNs aim at estimating the unknown positions of sensors by leveraging the known locations of a reduced set of nodes. Such a priori information is coupled with the availability of some position-related measurements such as TOA, time-difference of arrival (TDOA), received signal strength (RSS), or angle of arrival (AOA). In particular, among all the possible choices, RSS is very popular due to its ready availability and low complexity. Sensors with known positions are usually called “anchors”. Their locations are computed by resorting to the availability of GNSS receivers, or by deploying anchors at fixed known positions. Owing to different limitations regarding energy consumption, the specific environment at hand (e.g., GNSS is not available), and the impossibility of deploying nodes at desired positions (e.g., sensors are randomly scattered in the considered area), the position of most sensors is unknown. These nodes are typically called non-anchor nodes and their positions should be estimated within the WSN.

The widespread of sensor nodes in very different application areas led to a plethora of localization approaches for WSNs over the last years, which can be categorized according to four main groups as follows: (i) least squares (LS) methods; (ii) projection onto convex sets (POCS) methods; (iii) multihop methods; (iv) range-free methods. In the following, we briefly present some of them with the aim of providing a concise but sufficiently wide overview of traditional localization solutions in WSNs.

### 2.1. Least Squares (LS) Methods

One of the most common method to estimate the unknown locations of sensors in a WSN is based on LS and, more precisely, on the weighted LS (WLS) approach. Assume we are provided with a measurement vector $\hat{d}=d\left(x\right)+n$, which represents a set of (noisy) distance estimates carried out among nodes in a given WSN. The corresponding WLS estimate of position x of a specific node is obtained as

(1) $${\hat{x}}_{\mathrm{WLS}}=\underset{x}{arg min}{\left(\hat{d}-d\left(x\right)\right)}^{T}W^{-1}\left(\hat{d}-d\left(x\right)\right),$$

where W is a weighting matrix introduced to give greater weight to estimates that are deemed more accurate. In practical implementations, W is typically chosen as a function of the estimated covariance matrix. On the one hand, this method enables positioning capabilities even in very large WSNs, provided that a sufficient number of measurements $\hat{d}$ has been collected (e.g., TOA, RSS, AOA, or their combinations). On the other hand, the cost function in (1) represents a nonlinear optimization problem for which a closed-form solution is typically unavailable. In some cases, an approximate solution can be obtained by resorting to numerical optimization techniques. However, numerical routines suffer from the well-known issue of local minima, which may cause the optimization algorithms to converge towards a wrong solution. Furthermore, the numerical minimization of (1) involves a computational load that can become prohibitively high as the number of nodes increases. Hence, scalability and complexity are often the main issues with WLS-based position estimators.

### 2.2. Projection onto Convex Sets (POCS) Methods

Differently from the WLS estimator given in (1), POCS methods sequentially project points in a given space onto convex domains obtained from different sets of position-related estimates (e.g., distances, angles, …). The projection procedure is iterated until a point common to the intersection of all the considered sets is found. This point can be further refined by resorting to additional post-processing procedures (e.g., smoothing) or retained as an ultimate estimate of the node position. Examples of common geometries adopted for the convex sets include hyperbolas, ellipsoids, and cuboids. The main advantages of POCS-based approaches are the low complexity required to implement the estimators and the intrinsic simplicity to realize distributed solutions.

It is worth noticing that POCS-based methods are robust against the problem of local minima [56]. Furthermore, such methods can overcome the problem related to the overestimation of distances among nodes, as typical in TOA-based measurements subject to non-line-of-sight (NLOS) or multipath propagation phenomena [57]. On the other hand, the main disadvantage of adopting POCS methods is that they require nodes to be located within a limited distance from the anchor nodes. Indeed, when a node is located outside the perimeter defined by an anchor node, the intersection among the convex sets will be large and, as a consequence, the uncertainty on the position estimate will also be large.

### 2.3. Multihop Methods

In many real WSN deployments, nodes can typically interact only with a limited number of anchor nodes. Consequently, a very basic form of interaction among nodes is required to carry out the positioning process. Multihop localization algorithms have been proposed to this aim. A detailed comparison among different multihop methods is presented in [58], where authors outlined a three-step procedure common to most of the proposed algorithms. In the first step, nodes with unknown position information estimate their distances to anchor nodes in a multihop fashion. In a second step, each node employs some multilateration procedure (e.g., WLS) to obtain a coarse estimate of its position [59]. During the third and last step, each node refines its own position estimate by exploiting information about the neighboring nodes.

One of the most important methods, known as *N*-hop multilateration algorithm, was introduced in [60]. In this approach, nodes can approximate their distances to anchor nodes by adding the ranges measured at each separate hop. More precisely, the localization procedure starts when anchor nodes broadcast a first beacon over the network. This message contains, in addition to position and identity information, also a path length accumulator initially set to zero. Nodes that are able to receive the message can, in turn, estimate their range with respect to the previous node and add it to the path length accumulator. Then, they broadcast the updated message across the network. The main limit of multihop methods is that they tend to accumulate error at each hop; therefore, the ultimate distance estimate can be affected by a significant error. Multihop methods are preferable to other conventional approaches when operating in small WSN, and when an accurate hardware for range measurements is available. However, we again stress the fact that approximating LS cost functions as the one in (1) involves quite expensive operations, often not affordable in traditional low-cost nodes.

**Figure 2:**
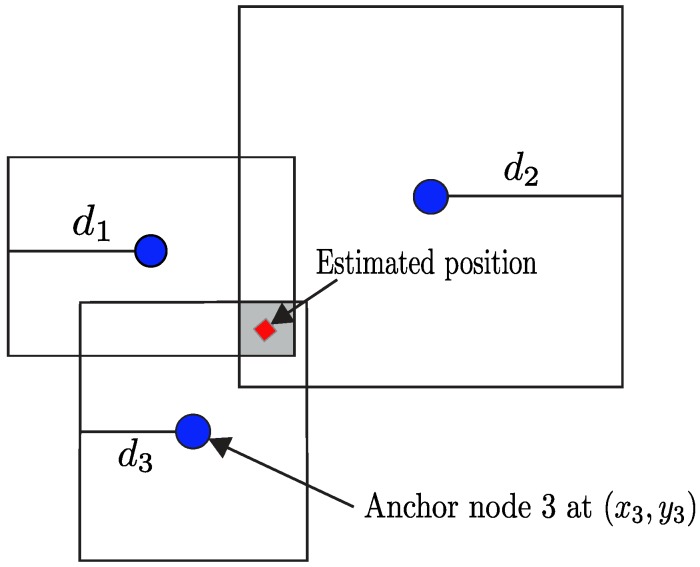
Min-max multilateration for the case of three anchor nodes.

Following the main idea behind the *N*-hop algorithm proposed in [60], a much simpler approach called min-max has been proposed. In this method, each node exploits the presence of anchor nodes to constrain its position estimates to be within a given range from each anchor. For the sake of illustration, a pictorial description of the min-max multilateration method is reported in Figure 2.

More specifically, if we assume that each node measures its distance $d_{i}$ to the *i*-th anchor node, then it can enforce its position to be within a bounded region whose coordinates are given by

(2) $$\left[x_{i}-d_{i},y_{i}-d_{i}\right]\times \left[x_{i}+d_{i},y_{i}+d_{i}\right],$$

where $\left(x_{i},y_{i}\right)$ denotes the known position of the *i*-th anchor node. The ultimate position estimate can be then restricted to lie within a more limited region, obtained as intersection among the set of bounded regions computed with respect to all the available anchor nodes, i.e.,

(3) $$\left[\max_{i}\left(x_{i}-d_{i}\right),\max_{i}\left(y_{i}-d_{i}\right)\right]\times \left[\min_{i}\left(x_{i}+d_{i}\right),\min_{i}\left(y_{i}+d_{i}\right)\right].$$

As a final step, each node selects the center of the region in (3) as its actual position estimate. The main advantage of min-max approaches is that they only require simple and low-complexity operations, hence can be implemented even in nodes with very limited computational capabilities.

Authors in [58] investigated the performance of the above discussed methods in terms of both positioning accuracy and coverage capacity. They considered a WSN deployment characterized by 225 nodes located in a squared area. Anchor nodes are the 5% out of the total number of nodes and are equally distributed over the network. Each node has an average connectivity of about 12–13 nodes and can interact with at least one anchor. Interestingly, it is shown that all methods exhibit almost the same sensitivity with respect to the number of anchors. Moreover, the approaches based on multilateration significantly outperform other competitors in presence of small ranging errors. The min-max method has been shown to be very robust to ranging errors, but it requires a careful deployment of anchor nodes. As a general conclusion, all the approaches exhibit different trade-offs between localization accuracy and coverage capacity.

### 2.4. Range-Free Methods

Another common way to infer location awareness is to exploit a rough information of proximity with respect to some reference points. Localization algorithms based on such a knowledge are typically called range-free. It is worth pointing out that, in the literature, the term range-free is also used to indicate localization techniques that directly exploit a statistical model to relate (typically in a highly non-linear fashion) the received raw signals to the unknown positions. However, such techniques are typically too complex for the limited processing capabilities of low-cost sensors in WSN. In the following, we will focus only on proximity-based approaches.

**Figure 3:**
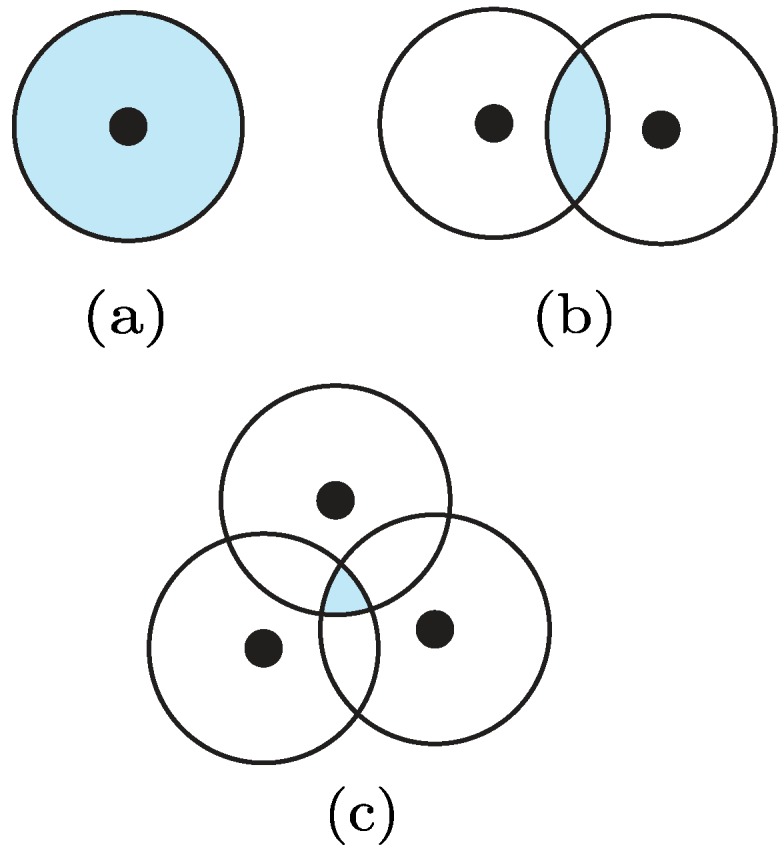
Region of possible solutions for $x_{n}$ as function of the number of proximity constraints.

We consider, as a reference scenario, a WSN deployed over a given area. Let us assume that *m* anchor nodes have been installed in fixed and known positions, i.e., $x_{a}={\left[\left(x_{1},y_{1}\right)\;\left(x_{2},y_{2}\right)\;\cdots \;\left(x_{m},y_{m}\right)\right]}^{T}$. In addition, assume that n−m nodes with unknown positions $x_{n}={\left[\left(x_{m+1},y_{m+1}\right)\;\cdots \;\left(x_{n},y_{n}\right)\right]}^{T}$ are present. The typical problem in this context is to estimate $x_{n}$ subject to a set of constraints derived from coarse proximity information. Proximity constraints are usually formalized through the well-known circular radio coverage model. In such a model, the limited transmission range of each node is represented with a circle having fixed radius *r*. For the sake of illustration, in Figure 3 we depict the set of possible solutions $x_{n}$ (shaded area) as function of the number of anchor nodes (black dots), namely m=1,2,3. As it can be seen, each proximity constraint contributes to a significant reduction in the size of the region of possible solutions for $x_{n}$.

Authors in [61] investigated the effect of deploying anchor nodes on a regular grid, separated by a known distance *d* from each other. Each node exploits the presence of the anchors in order to infer a set of proximity constraints. Interestingly, they demonstrated that higher values of the ratio r/d leads to much more accurate position estimates. Authors in [62] addressed the same problem by applying linear or semidefinite programming techniques. Remarkably, they noticed that the degree of network connectivity plays a key role in the positioning process.

The DV-Hop algorithm developed in [63] is one of the most important range-free positioning algorithms. Similarly to what happens in the *N*-hop multilateration technique, nodes exploit the coarse information contained in the number of hops to estimate their distance from each anchor. The localization process is supported by the anchor nodes which cooperatively compute the average single hop distance and broadcast it to all the other nodes. In doing so, each node can convert the number of counted hops into a meaningful (physical) distance from each anchor. In a final step, nodes apply a multilateration technique (e.g., min-max) to determine their position estimate.

Several algorithms have been proposed in literature to improve the performance of the DV-HOP approach. Just to provide some concrete example, authors in [64] derived a novel algorithm called weighted Monte Carlo localization (WMCL) based on a sequential Monte Carlo approach. By exploiting the position information provided by neighboring nodes within two-hop distance, the proposed WMCL can significantly reduce the average size of the bounding boxes up to 87%. As a direct consequence, the set containing the candidate position estimates shrinks, thus resulting in an improvement of the sampling efficiency up to 95%. Compared to the DV-HOP algorithm, the WMCL is able to provide a higher positioning accuracy while keeping both communication and computational costs at a reasonable level. In [65], a sequential Monte Carlo localization algorithm which exploits nodes mobility to achieve improved performance is devised. More precisely, each node dynamically updates a set of weighted samples representing its possible positions over time, subject to a constraint on the maximum allowed velocity. The final position estimate is then obtained by averaging only those samples that are consistent with the measurements obtained from the anchor nodes, while filtering out the remaining ones. The simulation analysis demonstrated that the proposed algorithm can achieve satisfactory localization performance even in presence of few anchor nodes and assuming a very limited amount of memory. An interesting fully distributed localization method called Orbit has been presented in [66]. This algorithm exploits additional connectivity-based constraints derived from the knowledge of the maximum range of coverage of each node. More precisely, nodes exchange their own range information on a cooperative basis and build a connectivity graph that can be used to reduce the set of feasible positions for each node. A first performance analysis has been conducted by means of simulations, also including a probabilistic communication model to reproduce realistic operating conditions; then, the algorithm has been tested on a real WSN deployment. The experimental results are in agreement with those obtained in the simulation analysis and showed that the proposed approach provides good localization performance, outperforming direct competitors under different configurations of the environment.

## 3. Localization and Tracking: Exploiting Sensor Mobility

In most of the current and previous generation of mote-class sensor networks, both anchor nodes, which have known locations, and non-anchor nodes have been assumed to be stationary and fixed. In the new era of crowdsensing networks, smart sensors are no longer static, but are rather characterized by an intrinsic mobility owing to the dynamic operating conditions in which they work (e.g., smartphones moving with people, in-vehicle sensors moving with cars, …). In this section, we depart from the traditional assumption of static sensors and review more recent localization techniques that can benefit from the presence of moving sensors.

### 3.1. Integration with Inertial Sensors

In the introduction of this paper, we have seen that the performance of GNSS-localized sensors can severely degrade in harsh environments such as (dense) urban areas or indoor scenarios. In such cases, in fact, the different sources of errors (e.g., multipath, shadowing, …) can cause a complete outage of the GNSS positioning service.

Recent research has shown that localization performance can be improved by leveraging on additional position-related information from other sensors (e.g., kinematic) available on-board. For instance, the measurements from smartphone built-in sensors (e.g., accelerometer, magnetometer, and gyroscope) can be fused together to estimate motion parameters such as speed, acceleration, orientation, etc. In such techniques, satellite positioning is integrated with inertial navigation [67,68], and nodes exploit only their own information, without interacting among themselves. For this reason, they are typically known as standalone methods.

The main idea consists of exploiting inertial sensors and the GNSS system in a complementary way. More precisely, inertial sensors can provide relatively accurate measurements (velocity, acceleration, …), but rapidly integrate errors over time owing to some intrinsic bias. On the other hand, a GNSS receiver is able to track nodes for longer periods, but it provides measurements that are typically affected by larger errors due to propagation effects from satellites to Earth. Furthermore, we recall that signals from satellites may be completely unavailable even in common environments environments (e.g., urban areas, indoor, etc.), thus causing an outage of the positioning system. In this section, we describe how both systems can be efficiently combined to produce a hybrid standalone approach able to improve their performance in those conditions when one system alone would fail to work, or would work with very poor performance.

In the literature [69], three conceptual approaches can be identified for integrating inertial navigation and GNSS-based positioning: (i) loose integration, (ii) tight integration, and (iii) ultra-tight integration. The main difference among these approaches consists of the way the information obtained from the two systems is jointly used as well as in the specific type of interaction among them.

An error-state Kalman filter (KF) approach, also known as complementary KF, is commonly used as an integration tool for all the considered methods. In such formulation, a node exploits the separated solutions provided by both inertial sensors and GNSS in order to compute a reference error [70]. In most practical applications, such an error is computed by subtracting the GNSS output from the predicted positions provided by the inertial sensors. The estimated error can be subsequently used to perform a correction in the inertial measurements. Mathematically speaking, the error-state KF is based on a state vector that accounts for the incremental differences among predicted and estimated values [71]. More precisely, the basic state vector $x_{k}$ is defined as

(4) $$x_{k}={\left[\Delta x_{k}^{e}\;\Delta v_{k}^{e}\;\Delta \psi_{k}^{e}\;b_{ak}^{b}\;b_{gk}^{b}\right]}^{T},$$

where $\Delta x_{k}^{e}$ is the incremental node position in the Earth frame, $\Delta v_{k}^{e}$ is the incremental node velocity, $\Delta \psi_{k}^{e}$ denotes the node attitude errors, and $b_{ak}^{b}$ and $b_{gk}^{b}$ are the biases affecting the accelerometer and gyroscope in the body frame, respectively. Additional states have been also been considered in the available literature [72]. Omitting the details, which can be found in [73], the state-space model can be formalized as

(5) $$x_{k+1}=F_{k}x_{k}+G_{k}w_{k},$$

where the transition matrix $F_{k}$ is given by [73]:

(6) $$F_{k}=\left[\begin{array}{ccccc} I_{3\times 3} & T_{s}I_{3\times 3} & 0_{3\times 3} & 0_{3\times 3} & 0_{3\times 3}\\ N^{e} & I_{3\times 3}-2T_{s}\Omega_{E}^{e} & -T_{s}\Phi_{k} & T_{s}C_{bk}^{e} & I_{3\times 3}\\ 0_{3\times 3} & 0_{3\times 3} & I_{3\times 3}-T_{s}\Omega_{E}^{e} & 0_{3\times 3} & -T_{s}C_{bk}^{e}\\ 0_{3\times 3} & 0_{3\times 3} & 0_{3\times 3} & I_{3\times 3}+T_{s}D_{a} & 0_{3\times 3}\\ 0_{3\times 3} & 0_{3\times 3} & 0_{3\times 3} & 0_{3\times 3} & I_{3\times 3}+T_{s}D_{g} \end{array}\right],$$

where $0_{N\times N}$ denotes the N×N matrix of zeros, $T_{s}$ denotes the sampling step, $\Omega_{E}^{e}$ is a skew-symmetric matrix accounting for the Earth turn rate, $N^{e}$ represents the centripetal and gravitational accelerations, $D_{a}$ and $D_{g}$ are the accelerometer and gyroscope models for bias, and $\Phi_{k}$ is a skew-symmetric matrix representing the type of measured forces $f_{k}^{b}$. Under standard hypothesis for the inertial sensors biases, the noise-state transition matrix $G_{k}$ is

(7) $$G_{k}=\left[\begin{array}{cccc} 0_{3\times 3} & 0_{3\times 3} & 0_{3\times 3} & 0_{3\times 3}\\ T_{s}C_{bk}^{e} & 0_{3\times 3} & 0_{3\times 3} & 0_{3\times 3}\\ 0_{3\times 3} & -T_{s}C_{bk}^{e} & 0_{3\times 3} & 0_{3\times 3}\\ 0_{3\times 3} & 0_{3\times 3} & T_{s}I_{3\times 3} & 0_{3\times 3}\\ 0_{3\times 3} & 0_{3\times 3} & 0_{3\times 3} & T_{s}I_{3\times 3} \end{array}\right].$$

As it can be noticed, different noise models lead to different KF formulations, hence to a different type of integration among GNSS and inertial systems, as will be clearer in the following.

We start by discussing the loosely integrated method. As the name suggests, this type of framework exploits the outputs obtained by using the inertial sensors and the GNSS receiver as separated navigation systems. More precisely, the KF observation vector is built by considering the difference between position and velocity estimates as produced by each independent system. It is worth highlighting that this method requires that both solutions are available at the same time. However, it is well-known that GNSS receivers have an acquisition rate much lower than the one of the local inertial sensors. Furthermore, GNSS-based navigation can suffer from severe outages (caused by bad environmental conditions) that may prevent its use even for long periods of time.

The observation equation for such a framework can be expressed as [69]

(8) $$y_{k}^{lo}=H^{lo}x_{k}+\nu_{k}^{lo}$$

where

- $y_{k}^{lo}=y_{k}^{sat}-y_{k}^{ins}$ is the measurement vector;
- $y_{k}^{sat}={\left[p_{k}^{sat^{T}}\;v_{k}^{sat^{T}}\right]}^{T}$ is the vector containing GNSS-based position and velocity estimates at time instant *k*;
- $y_{k}^{e}={\left[p_{k}^{e^{T}}\;v_{k}^{e^{T}}\right]}^{T}$ is the vector containing inertial-based position and velocity estimates at time instant *k*;
- $H^{lo}$ is the observation matrix for the loosely coupled framework, expressed as
  (9) $$H^{lo}=\left[\begin{array}{ccccc} I_{3\times 3} & 0_{3} & 0_{3} & 0_{3} & 0_{3\times 6}\\ 0_{3} & I_{3\times 3} & 0_{3} & 0_{3} & 0_{3\times 6} \end{array}\right];$$
- $\nu_{k}^{lo}$ is the measurement noise.

As it can be noticed, the loose integration scheme described by (5) and (8) is inherently linear. Therefore, the localization problem can be solved within the optimality conditions guaranteed by the KF.

In a tightly coupled framework, the localization performance can be improved by processing the information provided by both GNSS and inertial systems at the pseudorange level. Differently from what happens in a loosely coupled method, the KF is directly fed with an information that is somewhat proportional to the quality of the ultimate position estimates. Interestingly, this framework allows nodes to perform an integration even when only few satellites in view are available, while we highlight that at least four satellites are needed to compute a GNSS-based position estimate when resorting to loosely coupled methods. vIn the specific case of tight integration, the measurement vector can be defined as [73]

(10) $$y_{k}^{ti}=\zeta_{k}^{sat}-{\hat{\zeta }}_{k},$$

where

- $\zeta_{k}^{sat}$ is the vector of GNSS-measured pseudoranges $\rho_{k}$ and pseudorange rates ${\dot{\rho }}_{k}$ obtained at time instant *k*;
- ${\hat{\zeta }}_{k}$ is the vector containing the predicted pseudorange and pseudorange rates obtained from the last available estimate.

Therefore, we can write the observation vector as

(11) $$y_{k}^{ti}=H_{k}^{ti}x_{k}+\nu_{k}^{ti},$$

where $H_{k}^{ti}$ is a matrix expressing how the pseudoranges and the current states of the system are related to each other, while $\nu_{k}^{ti}$ denotes the measurement noise. In this case, the relation among positions and measured pseudoranges is non-linear, hence a linearization process should be applied, that is [74]

(12) $$H_{k}^{ti}=\left[\begin{array}{ccccc} U_{k} & 0_{N_{sat}\times 3} & 0_{N_{sat}\times 3} & 0_{N_{sat}\times 3} & 0_{N_{sat}\times 3}\\ 0_{N_{sat}\times 3} & U_{k} & 0_{N_{sat}\times 3} & 0_{N_{sat}\times 3} & 0_{N_{sat}\times 3} \end{array}\right],$$

(13) $$U_{k}=\left[\begin{array}{cc} -u_{1k}^{T} & 1\\ -u_{2k}^{T} & 1\\ \vdots & \vdots \\ -u_{N_{sat}k}^{T} & 1 \end{array}\right],$$

(14) $$u_{nk}=\frac{p_{k}^{n}-{\hat{p}}_{k}}{∥p_{k}^{n}-{\hat{p}}_{k}∥},\;\forall n=1,\ldots ,N_{sat},$$

where $p_{k}^{n}$ denotes the *n*-th satellite position, $N_{sat}$ represents the number of available satellites at time instant *k*, and ${\hat{p}}_{k}\overset{\mathrm{def}}{=}\hat{p}\left(t_{k}\right)$ denotes the last position estimate.

The ultra-tight integration framework is derived similarly to the tight integration one, but it considers as input to the KF filter both the in-phase and quadrature components obtained from the GNSS local correlators [75,76]. Also in this setup, the inertial-based measurements are corrected on the basis of the GNSS information. However, the specific type of information together with the particular correction applied to the position estimates are peculiar characteristics of the ultra-tight integration framework. It is interesting to observe that this approach provides better tracking performance and an improved robustness against the lack of satellites in view [77].

### 3.2. Hybrid RSS, TOA, and AOA Positioning

Filtering techniques as the ones discussed in the previous section allow nodes to track their positions and velocities when some dynamic aspect such as mobility comes into play, yielding a recursive framework that can be exploited to achieve more accurate estimation performance. To complete the overview of mobility-based positioning algorithms, in this section we present a common method to combine different types of terrestrial-based measurements, namely TOA, RSS and AOA, in a hybrid *GNSS-free* positioning system, based on the extended Kalman Filter (EKF) algorithm.

For the sake of explanation, we assume a 2D scenario, where a non-anchor node exploits the presence of several anchor nodes to localize itself. The *N* anchor nodes are available at known locations $p_{n}={\left[x_{n}\;y_{n}\right]}^{T}$, n=1,…,N. In the following, we assume that each node is equipped with different wireless devices (including an antenna array), providing TOA, RSS and AOA information. Thus, the observation vector $y_{k}\in R^{3N\times 1}$ at the time instant *k* is expressed as

(15) $$y_{k}={\left[y_{\mathrm{RSS},k}^{T}\;y_{\mathrm{TOA},k}^{T}\;y_{\mathrm{AOA},k}^{T}\right]}^{T},$$

while the related noise vector is given by

(16) $$\nu_{k}={\left[\nu_{\mathrm{RSS},k}^{T}\;\nu_{\mathrm{TOA},k}^{T}\;\nu_{\mathrm{AOA},k}^{T}\right]}^{T},$$

with time-invariant covariance matrix expressed as

(17) $$R^{\mathrm{hyb}}=\left[\begin{array}{ccc} R_{\mathrm{RSS}} & 0_{N\times N} & 0_{N\times N}\\ 0_{N\times N} & R_{\mathrm{TOA}} & 0_{N\times N}\\ 0_{N\times N} & 0_{N\times N} & R_{\mathrm{AOA}} \end{array}\right].$$

To mitigate potential biases deriving from the presence of NLOS propagation, the KF state is typically extended in order to include an additional unknown parameter that capture the shadowing effects experienced during the interaction with each anchor node [78]. In this respect, the new state vector is defined as $x_{k}^{\mathrm{hyb}}={\left[x_{k}^{T}\;b_{k}^{T}\right]}^{T}$ where $x_{k}={\left[p_{x}\left(k\right)\;p_{y}\left(k\right)\;v_{x}\left(k\right)\;v_{y}\left(k\right)\right]}^{T}$ is the vector of node position and velocity components at time *k* and the elements of the vector $b_{k}$ are the distance measurement bias at each anchor. Accordingly, the state transition matrix $F_{k}^{\mathrm{hyb}}$ can be written as

(18) $$F_{k}^{\mathrm{hyb}}=\left[\begin{array}{cc} F_{k} & 0\\ 0 & I_{N\times N} \end{array}\right],$$

where, assuming a nearly constant velocity model, the state transition matrix

(19) $$F_{k}=\left[\begin{array}{cccc} 1 & 0 & \Delta t_{k} & 0\\ 0 & 1 & 0 & \Delta t_{k}\\ 0 & 0 & 1 & 0\\ 0 & 0 & 0 & 1 \end{array}\right],$$

with $\Delta t_{k}\overset{\mathrm{def}}{=}t_{k}-t_{k-1}$. The additive process noise vector is given by $w_{k}={\left[0_{2}\;w_{s}\;w_{b}\right]}^{T}$, where $w_{s}$ and $w_{b}$ represent the velocity and bias noise vectors, and $Q_{s}$ and $Q_{b}$ denotes the corresponding covariance matrices. The exact expression of the measurement matrix for each different measurement can be found in [69]. The final matrix $H_{k}^{\mathrm{hyb}}$ can hence be obtained by concatenating the linearization matrices associated to each different measurement, that is,

(20) $$H_{k}^{\mathrm{hyb}}=\left[\begin{array}{c} H_{\mathrm{RSS},k}\\ H_{\mathrm{TOA},k}\\ H_{\mathrm{AOA},k} \end{array}\right].$$

Starting from the above mentioned models, each node can track its own position and velocity components by iterating the well-known KF prediction and update phases [69].

## 4. Cooperative Positioning

**Figure 4:**
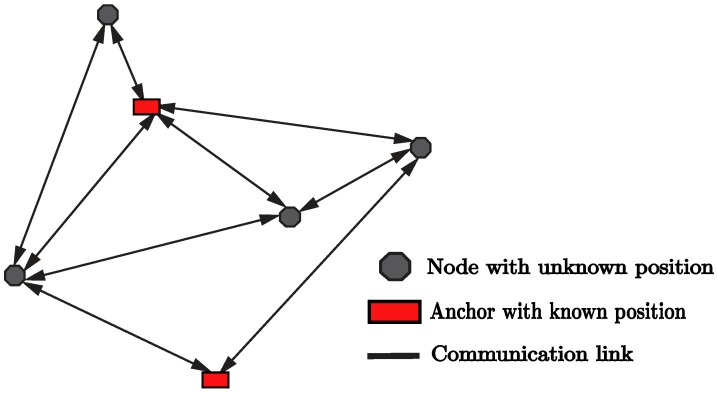
Principle of cooperative positioning.

Positioning algorithms in WSNs are required to satisfy stringent constraints in terms of energy-saving, robustness to hardware faults, and should also cope with different propagation phenomena at play in typical real-world deployments (e.g., NLOS propagation, multipath, interferences, …). As we have seen in the previous sections, in practical WSNs only few anchors can be installed in fixed and known positions, likely leaving most of the remaining nodes in isolated regions. In absence of anchors supporting the localization task, conventional methods adopted in traditional WSNs are not often able to provide a sufficient accuracy. To overcome such limitations, the positioning process can be performed *cooperatively*, i.e., by allowing nodes to exchange their own position-related information over the network, as shown in Figure 4.

The adoption of cooperative approaches lead to a number of important advantages: (i) large WSNs can benefit from the availability of a huge number of position-related measurements; (ii) the positioning task can be performed even in absence of anchor nodes; (iii) the availability of nodes equipped with different positioning systems (e.g., GNSS, 4G/5G cellular systems, and other terrestrial technologies) can have a substantial benefit over a cooperative framework. A prominent example of application of cooperative localization techniques can be found in vehicular ad hoc networks (VANETs), where cooperative positioning (CP) approaches have been considered in order to support the emerging road safety applications (e.g., overtaking-assistance, collision avoidance, etc.). Given the critical nature of such applications, a substantial research effort has been devoted in this direction from both academia and industry in recent years [79,80].

**Figure 5:**
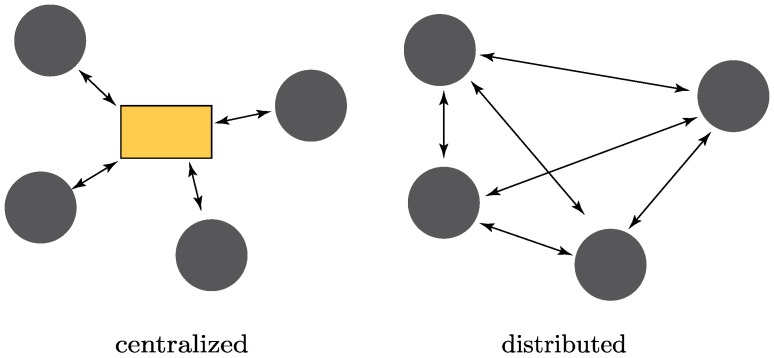
Centralized vs distributed cooperative positioning (CP) architecture.

CP methods can be broadly categorized in two main classes, namely centralized or distributed, based on the considered network architecture, as depicted in Figure 5. More precisely:

- **Centralized approaches:** in this paradigm, both anchor and non-anchor nodes send their collected measurements to a central (master) node, which is responsible for implementing the WSN localization algorithm that estimates the positions of all nodes in the network. The main advantage of this method resides in its simplicity. On the other hand, centralized approaches suffer from the critical risk of single point of failure. Furthermore, they typically require that the master node has high computational and networking capabilities.
- **Distributed approaches:** the WSN localization algorithms are executed over all the nodes, and the position estimate is computed locally. More precisely, nodes combine their own information with the measurements received from neighbour nodes in the network. In doing so, each node iteratively refines its own position estimate until a given criterion of convergence is reached. The main advantage of this paradigm lies in the reduced computational and communication complexity, which in turn lead to a significant energy saving. Indeed, distributed approaches avoid the burdensome operations required to send all collected data to a master node and receive position information back to all nodes in the network. This is a very important aspect, especially when dealing with large WSN deployments. Furthermore, distributed approaches do not require any fixed infrastructure, allow scalability, and foster the creation of time-varying WSNs having heterogeneous participating nodes.

The choice between centralized and distributed approaches is driven by several different aspects: (i) the type of network and sensors technology; (ii) the specific application area; (iii) the requirements in terms of response overhead; (iv) the limited autonomy of WSN nodes. From these points, it is evident that there exists a main trade-off among the computational capabilities of nodes and the maximum traffic load allowed in the network. It is worth pointing out that both distributed and centralized approaches solve the localization problem in a suboptimal way. This is clearly related to the the fact that they are subject to different types of constraints with respect to more traditional non-cooperative localization approaches.

In principle, CP techniques may extend traditional WSN localization algorithms by simply adding the dimension of the cooperation. A lot of research has been devoted to this topic; we refer the interested reader to [23,81,82] and references cited therein for a detailed overview. On the other hand, the design of novel CP solutions is also driven by various requirements related to the specific application areas. Just to give an example, a lot of effort has been put in the design of efficient CP techniques that can jointly satisfy all the typical constraints of low-cost WSNs. The problem of real-time node localization is also a critical aspect of many positioning systems. In this respect, the main challenge consists in adapting classical tracking techniques (e.g., Bayesian) to non-centralized (time-varying) topologies that should account for additional cost related to the exchange of information over the network [83]. Another important topic concerns the integration of multiple positioning systems in a unified CP solution able to provide anywhere and anytime location awareness.

In this section, we review the main CP paradigms where different position-related data and information are shared within nodes to enhance individual localization performance. In particular, the first devised CP strategy provided for the sole exchange of GNSS data. Clearly, this kind of approach can work only in presence of nodes equipped with a GNSS receiver. Examples of GNSS data are the measured pseudoranges, the number of visible satellites, the estimated position and velocity, and so on. Then, we move towards more recent CP methods which aim at combining heterogeneous position-related information coming from all the cooperative nodes available in the network. More precisely, they not only exploit the exchange of GNSS data, if available, but additionally consider information obtained from other systems (e.g., RSSs, TOAs, AOAs, inertial sensors, etc.). This hybrid approach, typically known as hybrid CP, requires that each node is equipped with a GNSS receiver and a proper hardware to estimate position-related data from the other nodes in the network.

### 4.1. Cooperation Based on GNSS-Only Information

**Figure 6:**
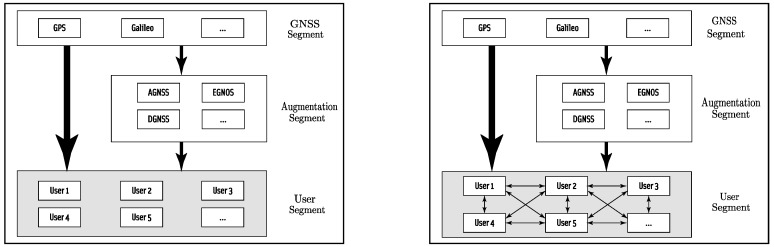
Non-cooperative vs. cooperative GNSS positioning.

GNSS-based navigation systems are characterized by information signals traveling from satellites to ground receivers. In order to compute a valid position estimate, each receiver should collect a minimum number of observations (e.g., four for a 3D localization) from satellites in view. To further improve the localization performance, additional ground-based augmentation systems have been developed. The standard GNSS scenario is represented in the left part of Figure 6. As multi-constellation and interconnected GNSS receivers will be soon available in the global market, GNSS cooperative positioning is expected to become a valid alternative to ground-based augmentation systems. The basic idea behind such approaches consists of observing that nodes in harsh operating conditions can exploit data received from other GNSS-equipped nodes in the network which are experiencing better satellite visibility. Then, they can apply their own positioning techniques for estimating their locations faster and with higher reliability, as shown in the right part of Figure 6.

One of the most promising approaches consists of exploiting exchanged raw GNSS observables in order to compute range estimates from other nodes [84]. These methods seem similar to the differential GPS (DGPS) approach, but they do not require the presence of fixed ground stations. According to the open literature, we can categorize GNSS data in two main groups: (i) carrier measurements and (ii) raw code [85]. Although carrier phase measurements are known to be very precise, they suffer from problems related to phase ambiguities and other intrinsic inefficiencies [86]. For these reasons, most of the research available in literature mainly focused on the adoption of raw codes [87,88,89]. Authors in [87] investigated the possibility to exchange GNSS code pseudoranges to estimate distances from neighbors and then subsequently used them within a particle filter (PF). Double difference (DD) of pseudoranges have been first proposed in [88] and later in [84] for static scenarios, then extended to mobile contexts in [89]. Since then, the adoption of DD of GNSS pseudoranges widespread across the CP framework [90,91].

### 4.2. Cooperation Based on Heterogeneous Information

It is well known that GNSS signals can suffer from severe blockages due to harsh propagation environments (e.g., urban canyons, indoor areas), thus resulting in a complete outage of the positioning service. A first approach to combat these drawbacks consists in exploiting information coming from on-board kinematic sensors within a standalone integrated system, as discussed in Section 3. However, we have seen that GNSS/Inertial systems tend to accumulate errors over time. Just to give an example, the lack of GNSS signal for a short period of 30 s would correspond to accumulated errors in the order of about 10–20 m for a node moving at 100 km/h. To solve the impasse, multi-system approaches that combine GNSS and terrestrial localization methods have been recently considered to improve both positioning availability and accuracy.

In this section, we focus on hybrid CP systems, where different information exchanged among heterogeneous nodes are used in the localization process. The main idea behind these techniques consists in exploiting the diffusion of position-related information made possible by the cooperation among nodes. Indeed, each node in the network can improve its position by jointly processing all the position-related data received from neighbors and combine them together with its own information through a proper estimation algorithm. This approach can provide significant improvements in terms of localization availability, even for GNSS-denied nodes; in fact, nodes having few satellites in sight may be able to compute their position by using terrestrial signals and neighbors information. Furthermore, nodes without any positioning capability can still be able to localize themselves by leveraging the information received over the network. In addition, hybrid CP methods do not require any dedicated infrastructure: each node performs its computation independently, starting from its own information and the messages received from neighboring peers. As a whole, such approaches are able to provide anywhere location capability, that is, in case of GNSS-only, terrestrial-only, and GNSS plus terrestrial available signals.

Different CP approaches have been investigated in recent years, mainly targeted to the automotive industry. Some CP schemes exploit the presence of terrestrial signals such as RSS, TOA, and TDOA in order to improve GNSS-based positioning [80,92], possibly combined with inertial information of nodes. For instance, authors in [80] addressed the problem of CP of a group of nodes by exploiting the exchange of RSS-based range estimates and the availability of local velocity measurements. Similarly, reference [92] proposed a novel EKF-based tracking algorithm fed with range estimates computed from TDOA measurements between nodes and GNSS position and velocity information. Interestingly, authors in [93] showed that the main limitation of such CP approaches resides in the insufficient information contained within the considered signals. More precisely, they proven that TOA/TDOA and RSS measurements cannot provide a sufficient accuracy, especially in harsh environments where the path-loss exponent cannot be properly estimated, and there are no simple methods to guarantee accurate synchronization among nodes. This issue can be very critical for CP algorithms that can leverage on the sole availability of terrestrial signals, as typical in urban or indoor scenarios [94,95,96]. In the following, we will show that the adoption of advanced signal processing techniques can bring significant advantages in the positioning process, even when dealing with more difficult operating scenarios.

## 5. Advanced Signal Processing for Localization and Tracking

So far, we have seen that the performance of most of the methods proposed in literature is limited by either the insufficient information content of the considered signals (RSS, TOA and TDOA) or the intrinsic limitations of the adopted technologies. In this section, we present a review of the latest results in the field of wireless positioning based on the adoption of advanced signal processing techniques. More precisely, we will show that PHY-layer plays a key role in providing useful position-related data to feed higher-level estimation algorithms.

### 5.1. Array Processing

Historically, the main disadvantages of adopting AOA-based localization techniques in low-cost WSN nodes were related to the fact that they required relatively large and complex smart antenna array. Nowadays, AOA-based methods are gaining momentum thanks to the widespread use of MIMO technologies in 4G/LTE cellular networks, and are becoming even more attractive for future 5G scenarios in which array size significantly shrinks, thus allowing such technologies to be integrated also in common mobile nodes (smartphones) [52].

In [97], authors proposed three novel tracking algorithms based on AOA measurements, the latter obtained from signals asynchronously received by several moving platforms. The dynamics of the nodes is modelled by resorting to the well-known interacting multiple models (IMM). Interestingly, although the three proposed solutions are only suboptimal, the achieved performance revealed a remarkable agreement with the theoretical lower bounds. Authors in [98] proposed a novel approach to track a single sensor by exploiting passive AOA measurements. The adoption of a track splitting algorithm together with a gaussian mixture model (GMM) for identifying the feasible node positions enable an accurate space-time tracking of the target. The AOA observations are fed to a bank of linear KFs as soon as they are available. Simulation results demonstrates the benefits of this approach, which is able to exhibit estimation errors falling to within 10% of the theoretical lower bounds. An accurate target tracking technique based on the adoption of beam-steering sensors is presented in [99]. At each step of beam steering, the node computes a rough estimate of the target location by exploiting the presence of the closest anchor node. Each sensor is responsible to track the target node until it pass by the field of view (FoV) of the passive sensor. The algorithm performance has been analyzed for different mobility models of the target. Interestingly, the proposed approach can effectively track a target moving at speeds that range from a minimum of 3 m/s up to a maximum of 8 m/s.

AOA-based localization approaches have also been considered in the context of indoor localization. In [100], authors proposed a fine-grained WiFi based localization system called ArrayTack which exploits the availability of eight-antenna arrays to track users moving inside buildings. Such an approach is able to distinguish the direct LOS path from the multipath reflections and is able to achieve an accuracy of below 1 m in typical indoor scenarios. Another interesting method which does not require any dedicated device has been derived in [101]. The algorithm leverages the multi-dimensionality of WiFi signals to passively track multiple moving people. In addition, a path separation procedure is employed to resolve multipath propagation. The experimental evaluation conducted by implementing the proposed method on both commodity WiFi devices and software define radio platform demonstrated that an average accuracy of 47 cm can be achieved. Authors in [102] presented a robust localization approach that exploits WiFi channel state information to refine the AOAs estimated from NLOS paths. The method uses a background elimination algorithm and the availability of TOF measurements to remove the NLOS paths from the target reflected signals. After this preliminary step, the final position estimation is performed by resorting to a particle filter. The experimental results revealed that the algorithm can provide sub-meter accuracy in two different indoor scenarios. In [103], a novel localization system called Phaser is proposed. By exploiting the availability of phased antennas on modern WiFi access points (APs), such an approach is able to adaptively calibrate itself and to mitigate the effects induced by multipath reflections. The experiment campaign conducted using an AP equipped with three and five antennas showed that the proposed approach is able to achieve improved calibration and localization performance. An interesting AOA-based approach to localize interfering sources has been presented in [104]. It exploits cyclostationary features of the signals measured at different APs to discriminate the signal transmitted by each interfering station. Differently from most of the previously discussed approaches, it requires a very limited calibration during the deployment phase. The algorithm has been validated using WARP software defined radios and the obtained results showed that it can achieve an average localization accuracy of 0.97 m. A novel device-free positioning system to track human movements inside buildings has been proposed in [105]. The main idea consists in exploiting the backscattering signals originated from common WiFi transmissions in indoor environments to tracing the motion of the reflecting objects. To this aim, a sequential estimation test is designed which provides a prediction of the most probable trajectories based on the estimated reflection parameters. Preliminary experiments have been conducted by implementing the proposed approach on low-cost software defined radios. The obtained results showed that it can provide good localization performance even in presence of multiple concurrent human motions.

Another interesting research direction has been investigated in [106], where AOA estimates—computed by applying standard estimation algorithms [107] to beacon packets broadcast by anchor nodes—feed a novel WLS positioning algorithm. The obtained performance revealed that the proposed WLS estimator is able to exploit the presence of fixed geolocated nodes to significantly increase the quality of the position information, with a resulting improvement of about 70% compared to plain GNSS. The same idea has been extended within a recursive estimation framework in [108] where a more general scenario which additionally considered the cooperation among a selected set of mobile nodes has been addressed. Remarkably, the simulation analysis demonstrated that the proposed EKF-based CP algorithm can achieve a position accuracy closer to the one of the most accurate node exploited in the network. An alternative localization algorithm based on a change-detection approach to localize nodes moving in transversally-bounded regions (e.g., roads, rails, corridors, etc.) has been presented in [109]. In such a method, nodes exploit the time-varying nature of AOA estimates collected along their trajectory to formalize a statistical hypothesis test aimed at identifying the time instant at which they cross a node (fixed or mobile). The detected events are then combined with local velocity measurements to improve the localization performance.

The main advantage of using AOA information is that, similarly to RSS measurements, it does not require any preliminary synchronization. Moreover, when used for multilateration, the number of AOA measurements required to localize a node are less than that required in case of TOA/TDOA or RSS measurements. To exploit AOA information, the only additional requirement is that nodes should be equipped with a multi-antenna receiver, leading to the so called single-input multiple-output (SIMO) systems. However, directional information has proven to be very valuable for positioning, hence can be regarded as a strong candidate for the design of next-generation localization systems where, as will be clearer in next sections, multiple antennas will be likely available at both transmitter and receiver sides.

### 5.2. Direct Position Estimation

The problem of node localization in dense multipath environments is a challenging and hot topic in signal processing. It usually arises when the node operates in the presence of obstacles (e.g., walls, trees, etc.) that cause the transmitted RF signals to be received via many propagation paths. In such conditions, typical of indoor and urban areas, traditional approaches like the ones described in previous sections, commonly known as indirect position estimation (IPE) approaches, may fail in providing reliable position information. In this section, we review the problem of node localization in multipath environments by resorting to a direct position estimation (DPE) approach. Contrary to traditional techniques, the key idea consists in determining the location of the node directly from the raw signals, without estimating any intermediate position-related parameter (RSS, TOA, AOA). In doing so, the constraints between the collected measurements and the node positions are explicitly included in the position estimation, resulting in a significant improvement of the achievable performance [110,111].

Direct localization approaches were first derived in [112,113], then applied to localization based on AOA measurements [114] and recently, also to hybrid AOA/TOA positioning [115,116]. In static scenarios, ML DPE has been developed for a single-path channel where multipath phenomena are modeled as additive noise [115,117], then extended to multiple nodes localization [116]. A novel DPE approach based on the principle of minimum-variance distortionless response has been proposed in [118,119]. Simulation results demonstrated that it has better resolution and immunity to jamming and interference, but its accuracy tends to degrade at low SNRs. DPE methods tailored to special signals such as OFDM, cyclostationary signals, and intermittent emissions have also been proposed [120,121,122]. In [123] a ML direct location estimator using wideband signals for nodes in the near field of the sensor array is derived. In [124], authors proposed a novel DPE method able to work even in presence of dense multipath. The algorithm is based on the ML principle and exploit TOA information. The main drawback is that it requires some a priori information about the channel, and is limited to localization of a static node. A novel TOA-based direct localization technique for multiple nodes leveraging on ideas from the compressive sensing theory has been recently proposed in [125]. In [126], a novel DPE algorithm for dense multipath scenarios under mobility is derived. The proposed approach has low complexity and is fully adaptive, i.e., it does not require any tuning or additional information about the environment. The algorithm employs an adaptive beamforming technique to reconstruct an estimate of the optimal projection matrix, which is then used to project the received signal over the suitable directions that maximize the likelihood. Furthermore, it takes advantage of a simple and effective LOS association method to identify the most probable node initial position, hence achieving an integration gain over time. The performance assessment revealed that the approach is very effective in (severe) multipath conditions, significantly outperforming natural competitors also when the number of antennas and snapshots is kept at the theoretical minimum.

As mentioned, with the widespread of MIMO technologies, AOA-based DPE algorithms are gaining momentum; in particular, large arrays enable a precise estimation of multipath parameters thanks to their high angular resolution. A novel ML estimator able to localize a single node in presence of static and known multipath has been proposed in [127]. Although the idea seems interesting, there is no efficient way to compute the estimator. Recently, a novel algorithm called direct source localization (DiSouL) has been proposed [128]. It is based on ideas from the compressed sensing theory and exploits some peculiar property of the channel in order to discriminate LOS from NLOS signal paths, leading to superior performance compared to other approaches. However, at more conventional radiofrequencies (cmWave), the multipath is not always resolvable; moreover, standard AOA estimation approaches are inapplicable because of the coherence among the received multiple paths that originate from the same signal.

### 5.3. Millimeter-Wave (mmWave) Technology

The millimeter-wave (mmWave) band is currently regarded as the leading technology for next generation wireless systems [129]. Differently from traditional systems that operate at frequencies below 6 GHz, the mmWave technology considers carriers with frequencies ranging from 30 GHz to 300 GHz. The main advantage of moving towards higher frequencies resides in the availability of larger channels. Just to give a concrete example, channels with 2 GHz of bandwidth are common for systems operating in the 60 GHz unlicensed mmWave band. Such a technology is also receiving tremendous interest by academia, industry, and government as a main building block for future 5G cellular systems [130,131,132]. To provide improved communication performance, most mmWave systems will be equipped with large antenna arrays at both the transmitter and receiver sides, thus creating many opportunities to exploit the benefits of MIMO communications. Large antenna arrays, in turn, enable the design of extremely narrow beams which can precisely point towards a specific direction, thus allowing accurate spatial resolution in the angular domain [133,134]. The connection between (massive) MIMO and mmWave paves the way for innovative mmWave MIMO systems, which are expected to provide unprecedented tools for precise (cm-level) localization thanks to their high temporal resolution and high directivity.

For the specific case of 2D geometry, a mmWave MIMO channel model can be generally expressed as

(21) $$H\left(t\right)=\sum_{i=1}^{M}H_{i}\delta \left(t-\tau_{i}\right),$$

where *M* denotes the total number of paths (including the LOS link), $\tau_{i}$ is the TOA associated to the *i*-th path and

(22) $$H_{i}\overset{\mathrm{def}}{=}\sqrt{N_{r}N_{t}}\beta_{i}a_{r,i}\left(\theta_{r,i}\right)a_{t,i}^{H}\left(\phi_{t,i}\right)\in C^{N_{r}\times N_{t}},$$

with $N_{r}$ and $N_{t}$ number of receiving and transmitting antennas, $\beta_{i}$ complex gain of the *i*-th path, $\theta_{r,i}$ and $\phi_{t,i}$ the AOA and angle-of-departure (AOD) of the *i*-th path, respectively. As concerns $a_{t,i}(\cdot )\in C^{N_{t}}$ and $a_{r,i}(\cdot )\in C^{N_{r}}$, they denote the (unit-norm) array steering vectors of the *i*-th path at the transmitter and receiver, respectively, and depend on the specific arrays structure [135]. From Equations (21) and (22), it can be observed that several position-related parameters are available in mmWave MIMO scenarios. The estimation of channel parameters such as RSS, TOA, AOA, etc., has been largely addressed in conventional wireless networks as starting point to perform positioning via multilateration techniques. Most of the available methods typically exploit some correlation metric between the received and expected signals in order to extract AOA or TOA information [107,136]. Unfortunately, in many cases of interest, multipath components arrive at the receiver with similar AOAs or TOAs, that is, they overlap in either the time or space domains. In such situations, the classical methods for AOA and TOA estimation are no longer optimal [135,137]. Conversely, in mmWave channels, the number of multiple paths *M* in (21) is typically small and corresponds only to single-bounce reflections [138]. For this reason, mmWave channels are considered spatially sparse, that is, all the parameters of different paths can be assumed to be distinct. Furthermore, as the system bandwidth increases, the multipath components can be more easily resolved in the time domain. In other words, all the multiple paths can be considered to be orthogonal [139,140], thus reducing the parameter estimation to a problem of multiple single-path estimation. In this respect, mmWave MIMO techniques brings the following benefits:

- while conventional methods cannot distinguish two signals arriving from the same direction, mmWave-based techniques can separate such contributions by exploiting their delays;
- since more parameters can be exploited in mmWave MIMO channels (namely AOA, AOD, and TOA), position of nodes can be estimated even in presence of a single anchor;
- mmWave has the ability to turn NLOS propagation, traditionally a pitfall of wireless transmission, into a benefit for the positioning process.

The theoretical localization performance achievable using mmWave MIMO have been recently investigated in [141,142,143]. In [141], the Cramér-Rao lower bound (CRLB) on the position and rotation angle estimates obtained using mmWave from a single transmitter has been derived. Furthermore, a novel position and rotation estimation algorithm based on compressed sensing that attains the CRLB for average to high SNR is proposed. In [143], authors investigated the fundamental limits of position and orientation estimation for uplink and downlink channels. Authors in [142] have shown that NLOS components can also be exploited to gain additional information for the positioning task. Despite the importance of this growing field, a few papers have proposed more sophisticated localization schemes, trying to take advantage of the peculiarities of mmWave and (massive) MIMO technologies. In [144], a 3D indoor positioning scheme based on hybrid RSS and AOA, which employs only a single BS has been presented. A hypothesis testing localization approach is proposed in [145] exploiting the concept of channel sparsity. A low-complexity AOA-based approach with signal subspace reconstruction is devised in [146] to localize incoherently distributed sources. An EKF tracking algorithm that jointly exploits AOA and TOA from uplink reference signals has been proposed in [147]. Authors in [148] addressed the problem of positioning based on joint TOF, AOD, and AOA estimation and investigated the impact of errors in delays and phase shifters. A hybrid TDOA, AOA, and AOD localization scheme is presented in [149] based on linearization of a set of local constraints, while in [150] positioning is addressed using a Gaussian process regressor based on a fingerprinting technique operating on a vector of RSS measurements. Another interesting method for the joint estimation of node position and orientation in 5G systems has been devised in [151]. In this work, authors exploit the sparsity nature of mmWave channels and adopt a compressive sensing with iterative refinements to accurately estimate AOAs, AODs, and TOAs of all the observed paths. Based on such estimated parameters, the unknown position and orientation of the target as well as the scatterers position can be described by resorting to an iterative Gibbs sampling-based approach. The simulation analysis highlights that the proposed approach can achieve cm-level position accuracy even in absence of a LOS path. The problem of tracking position and orientation through mmWave MIMO systems has been recently addressed in [152]. In this work, authors derive a channel training method based on the availability of lens antenna arrays. The channel estimation problem is formulated separately in both uplink and downlink following a sparse signal recovery representation. The obtained results revealed that the proposed approach can achieve superior performance compared to the compressed sensing solutions proposed in [141].

The potential benefits of mmWave communications have also been investigated in [153] for the problem of accurate vehicles localization. In this work, authors analyze how the ultimate localization accuracy is affected by the presence of multipath and clock biases. Interestingly, they demonstrated that the additional geometric constraints introduced by multipath propagation can be fruitfully used to solve the position estimation problem. The validity of such findings has been further confirmed in [154], where authors present a generic localization approach based on downlink signals transmitted by a single BS. The same idea has been then extended to the problem of vehicle tracking in [155] based on a Bayesian filtering approach. Authors in [156] presented a novel mmWave-based localization algorithm called mTrack. It leverages both RSS and signals phase measurements as main features to estimate the unknown object relative angle with respect to the a transmitter in known position. Such information is subsequently used to perform a tracking of the object over time. The experimental results revealed that mTrack can provide 90% of errors below 8 mm for a fine-grained pen tracking in a small office scenario. The 60 GHz mmWave band has been also considered in [157] to address the problem of mobile radar imaging. The proposed approach exploits a set of RSS measurements collected along the object trajectory to reconstruct its main surface features (including curvature, orientation and boundaries) with high precision. The whole system has been tested on real 60 GHz beamforming radios. The obtained results showed that the proposed localization approach achieves high accuracy and is robust against noises in device position and trajectory tracking.

The mmWave technology has been recently adopted also in the context of simultaneous localization and mapping (SLAM). Strictly speaking, SLAM consists of constructing and updating a map of a specific unknown environment while simultaneously tracking the positions of a node moving within it. Authors in [158] addressed the interesting problem of joint localization and mapping within the emerging paradigm of mmWave 5G systems. The main idea consists in exploiting the peculiarities of mmWave channels to gain additional information also from NLOS propagation. To this end, ref. [158] proposes a message passing-based estimator that uses a non-parametric belief propagation approach to obtain the unknown target position and orientation, as well as to determine where reflectors and scatterers are located. The numerical analysis shows that the proposed estimator provides high localization accuracy even in case of obstructed LOS propagation, without requiring any a priori knowledge of the environment.

Another interesting approach has been proposed in [159], where authors addressed the problem of mobile node localization within a multiple-input single-output (MISO) system. This setup is motivated by the fact that massive arrays will be initially implemented only on base stations, likely leaving mobile nodes with one antenna. While conventional SIMO localization schemes mainly focus on AOA estimation, the proposed algorithms aim at exploiting the AOD of received downlink signals, which can be estimated using a single ominidirectional antenna, thus avoiding the high computational cost required by large arrays. The obtained performance demonstrated that mmWave and MISO are enabling technologies for designing accurate positioning systems that can be readily implemented in the near future.

## 6. Conclusions

The advent of the digital era is driving the shift from the concept of mote-class static sensors to the modern concept of smart devices, paving the way for a new type of wide-area WSN, built on the top of existing communication technologies and based on massive crowdsensing of heterogeneous data. Within this novel framework, the provision of ubiquitous and accurate position information is of vital importance for a variety of emerging applications in the personal, social, and public dimensions. Compared to the state-of-art of WSN localization, further research is needed to accommodate the requirements imposed by emerging crowdsensing applications, especially under mobility and harsh environments typical of urban and indoor scenarios. In this survey, we reviewed recent advances in the field of wireless positioning, with specific focus on novel localization solutions that can cope with the peculiarities of crowdsensing WSNs; in particular, we have shown that the combination of mobility exploitation, cooperation, and advanced signal processing is a promising approach for the design of accurate next-generation positioning systems in such a context.
